# The importance of “being seen”: a qualitative interview study of patient and clinician experiences of virtual and face-to-face TIA clinics in one region of England

**DOI:** 10.1186/s12913-026-15470-6

**Published:** 2026-09-14

**Authors:** Jacob Heath, Carol Dumelow, Maeve Moran, Sara E. Shaw, Sarah Brown, Gary A. Ford, Catherine Pope

**Affiliations:** 1 Medical Sociology & Health Experiences Research Group, Nuffield Department of Primary Care Health Sciences, University of Oxford, Oxford, UK; 2 Trinity College Dublin, Dublin, Republic of Ireland; 3 Interdisciplinary Research in Health Sciences, Nuffield Department of Primary Care Health Sciences, University of Oxford, Oxford, UK; 4 Department of Psychiatry, University of Oxford, Oxford, UK; 5 Radcliffe Department of Medicine, University of Oxford, Oxford, UK

**Keywords:** TIA, Minor stroke, Virtual clinic, Remote, Telemedicine, Hybrid, TIA pathway, TIA mimic

## Abstract

**Background:**

Most NHS trusts introduced virtual clinics for managing transient ischaemic attack (TIA) during the Covid-19 pandemic. Some continue to use virtual clinics, whilst others returned to face-to-face or implemented hybrid clinics. There has been limited evaluation of the effectiveness and experience of patients and healthcare professionals (HP) of virtual TIA clinics.

**Methods:**

A rapid insights evaluation of the effectiveness of virtual TIA clinics took place in South East England. This paper focuses on findings from one part of the evaluation, which conducted qualitative interviews with 15 patients and 12 HPs across 6 NHS trusts to explore their views and experiences of different TIA clinic care models. Patient participants were identified by clinicians at first consultation with the TIA clinic. Telephone or video interviews were recorded and analysed using thematic analysis.

**Results:**

HPs reported that virtual clinics worked well for some patients (e.g., those not needing MRI/MRA imaging, older patients with co-morbidities/poor mobility, young working people, people living in rural areas) but not all. Most patients placed great importance on ‘being seen’ by a HP in a face-to-face consultation during their TIA care pathway. We identified two key themes which represent different yet complementary dimensions of the importance of ‘being seen’, highlighting the perceived value of face-to-face consultations over virtual TIA clinics: (1) Greater opportunities for communication; and (2) Feeling looked after and listened to by a multidisciplinary team.

**Conclusions:**

Virtual TIA clinics were perceived more positively by HPs than patients, who particularly valued face-to-face consultations. Yet HPs also identified distinct benefits of face-to-face consultations. Whilst further research is needed, our findings indicate that TIA service model design should consider how HPs interact with patients during assessment and diagnosis. A hybrid model of TIA clinic care may offer the greatest potential benefits to patients and clinicians. Offering choice of TIA clinic modality could be a priority, along with improved referral that reflects which patients are best suited to virtual or face-to-face care.

**Supplementary Information:**

The online version contains supplementary material available at https://doi.org/10.1186/s12913-026-15470-6.

## Background

To help limit the spread of Covid-19, efforts were made to shift as much of UK NHS primary and secondary care as possible to remote delivery, driving a push for ‘remote by default’ (we consider ‘remote’ and ‘virtual’ as synonymous when discussing telemedicine in TIA care consultations [1, 2] and use ‘virtual’ to align this paper with our related report [3]). By July 2020, the UK government advised that the majority of outpatient consultations should be teleconsultations [4]. Medical specialities had to rapidly adapt to new ways of delivering healthcare as a result. The Oxford Academic Health Science Network / ‘Getting It Right First Time’ (GIRFT) report published in May 2020 provided guidance on rapidly adapting stroke services to accommodate Covid-19 safety guidelines, recognising that stroke management practice would have to quickly change [5].

The policy drive for digitising UK healthcare had been underway long before the Covid-19 pandemic began, with digital technologies proposed to improve the quality and reduce the cost of healthcare [6–8] leading to initial studies examining its advantages and limitations within secondary care [9, 10]. A 2018 report by the Royal College of Physicians proposed that digital services be offered in reorganising outpatient care, with a continuing need for traditional face-to-face services for those who cannot/will not engage with digital technologies [11]. A year later, the ‘NHS Long Term Plan’ [12] emphasised the need for a shift to digitized primary and outpatient care, whilst in 2020 virtual forms of healthcare were figured into the NHS’ future through long-term plans to reduce its carbon emissions to net zero [13]. Now, virtual forms of healthcare delivery (e.g., phone, video and web-based services) are widely established and often referred to as the “new normal” [14].

The widespread adoption of virtual care has been accompanied by growing recognition that digitised forms of healthcare are not necessarily beneficial for everyone, cannot be used for everything, and are variably implemented by service providers [15–17]. There remains a tension between providing a sustainable resource-efficient healthcare service and offering patient choice [9, 18]. The PERCS (Planning and Evaluating Remote Consultation Services) Framework has been helpful in highlighting that remote consultations are complex, with many interacting factors in need of consideration to ensure high-quality consultations and care [19]. For example, it has been argued that notwithstanding ‘on the job’ experiential learning during the pandemic, clinicians need better training to deliver virtual care [19–21]. There is also a need to consider the materiality of the technology used to deliver remote care, how it mediates the patient-clinician relationship, and the ways that virtual modalities potentially shape interaction [22, 23]. Therefore, identifying which forms of patient consultation can function virtually without compromising on quality, whilst also considering patient preferences for mode of consultation, is crucial [16].

### Virtual care for patients who have experienced a stroke or TIA

Some stroke specialists have identified a loss of cues in virtual consultations in comparison to face-to-face consultations, as well as finding that learning to trust staff skills of virtual consultations took time [24, 25]. This is not unique to stroke services, reflecting wider literature on virtual care [25]. Where patient perspectives on virtual stroke services have been explored, face-to-face consultations were preferred and perceived to be a better mode of delivering care, despite high levels of satisfaction with stroke telemedicine consultations [26, 27]. These sentiments echo wider literature on virtual care and consultation, which reveals NHS patients can be satisfied with virtual services yet still perceive face-to-face as superior and preferable [28].

A study that examined virtual care in TIA outpatient clinics has reported positively on short-term clinical outcomes for patients and for the management of patient care [29]. But there is limited evidence on patient and clinician experiences with virtual TIA clinics, which usually utilise telephone consultations as patients are seen within 24 h of referral making video calls difficult to set up. This paper contributes to research on patient and clinician experiences of virtual clinics for TIA, reporting on findings from one part of a larger rapid insights evaluation of the effectiveness of virtual TIA outpatient clinics in the South East Region of England [3].

## Methods

### Study design

The overarching rapid insights evaluation [3] informing this paper was supported by the NHS Insights Prioritisation Programme (NIPP). NIPP was launched in 2021 to accelerate the evaluation and implementation of innovations that support post-pandemic ways of working, build service resilience and deliver benefits to patients. Different TIA service models including virtual, face-to-face, and hybrid clinics were evaluated to produce a ‘rapid insights’ guide for healthcare staff, service providers, and NHS commissioners. The evaluation [3] sought to highlight opportunities for quality improvement, incorporating learning from the pandemic and considering health inequalities, digital exclusion, and environmental impact. The evaluation focused on 26 TIA out-patient services clinics within 5 Integrated Stroke Delivery Networks (ISDNs) in South East England and comprised 4 workstreams; pathway mapping, views of patients and health care professionals, sustainability and use of resources.

This paper focuses on the findings from the views of patients and healthcare professionals workstream. Drawing on data from interviews with patients attending TIA clinics (who were being investigated for a suspected TIA) and healthcare professionals, we sought to understand their views and experiences regarding virtual, face-to-face and hybrid models of TIA outpatient care. As the patients and healthcare professionals we interviewed may not have experienced or delivered each model of TIA outpatient care, the aim of these interviews was to explore the perceived benefits and disadvantages of each of the three models.

### Setting and context

#### Setting

**Fig. 1:**
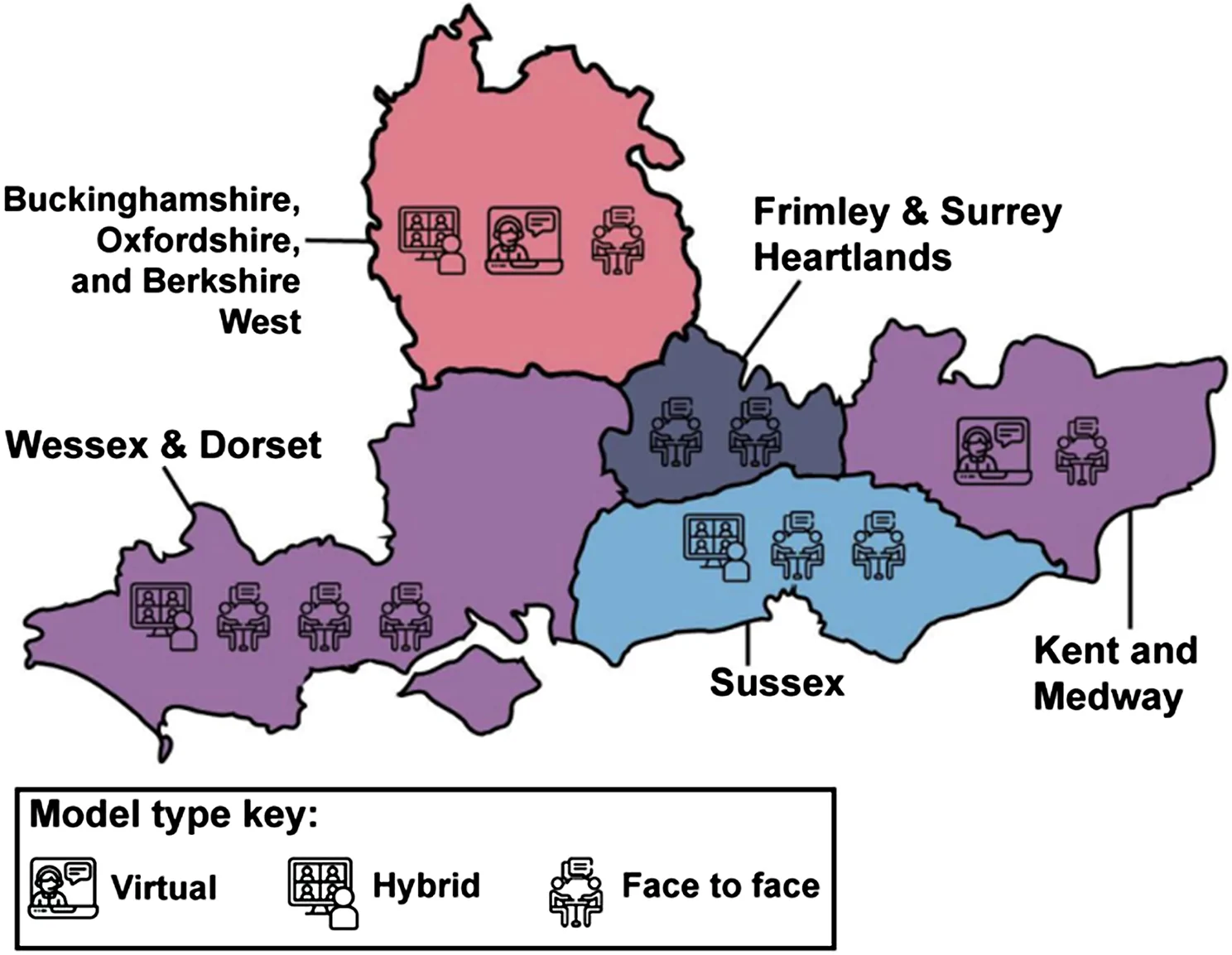
Number of south east services using each model by integrated stroke delivery network (ISDN)

At the time of the evaluation, the South East Region of England was divided into 5 ISDNs (Fig. 1), with TIA outpatient clinics operating in each. In some ISDNs, car travel to the TIA clinic could be over an hour. The main purpose of TIA clinics is to enable a stroke specialist to make a diagnosis, undertake appropriate investigations and initiate treatment in patients referred to them by their GP or from emergency services, as a result of the patient experiencing suspected TIA symptoms. Many patients referred to a TIA clinic are diagnosed with a TIA mimic, an episode of symptoms similar to those of a TIA but specialist assessments determine other causes, for example migraine with aura [30, 31]. NICE guidance states patients with a suspected TIA that has occurred within the last week should be assessed by a stroke specialist within 24 h of presentation to primary care [32].

### TIA care pathway – face-to-face, virtual and hybrid

After the Covid-19 pandemic, most TIA services adopted a hybrid model, but some returned entirely to face-to-face and a few remained fully remote. There is also significant variation within each service model design. To understand the TIA Care Pathway in the South East Region, a pathway mapping was therefore conducted which led to the development of definitions for the study [3]. We use these definitions in this paper:

*Face-to-face model* – most patients are seen in person for their TIA clinic appointment.

*Virtual clinics* – most patients’ appointments and consultations are completed by telephone, or occasionally video call, with patients only coming into hospital for an MRI scan or other tests.

*Hybrid clinics* – a blended approach of the above, dependent upon patient and service need.

### Sampling and recruitment

We used purposive patient sampling, working with NHS TIA clinics in the five ISDNs of the South East region of England to select patients, aged 18+, who had experience of attending a TIA clinic between February and May 2023, for the research team to approach for interview.

Clinics in six trusts identified patients from TIA clinics and were encouraged to include participants from groups experiencing poorer health outcomes and to ensure that geographic (inner city, urban, rural), ethnic, and socio-economic diversity were represented. Patients were asked to contact the research team if they were interested in taking part in an interview. At their first consultation, patients received information about the study and were asked to contact the research team if interested in taking part.

Healthcare professionals (consultant stroke physicians and neurologists, advanced clinical practitioners, specialist stroke nurses, and clinic coordinators/administrators) who were involved in delivering TIA clinic care (in the same South East region) through virtual, face-to-face, or hybrid models of care between February and May 2023 were invited to participate in study interviews via email, in addition to snowballing through existing professional and research network contacts, and through the South East NHS region clinical stroke lead meetings.

### Data collection

15 patients and 12 healthcare professionals spread across the South East region were interviewed (Table 1). A roughly equal number of patients had attended a virtual clinic or a face-to-face clinic (Table 2) following referral from their GP, or from A&E, as a result of experiencing suspected TIA symptoms between February and May 2023. Patients were interviewed shortly after initial consultation, imaging and diagnosis.

**Table 1:** Spread of participants across south east region

ISDNs in SE region	Patients interviewed *n* = 15	HPs interviewed *n* = 12	Total *n* = 27
Frimley & Surrey Heartlands	4	2	6
Wessex & Dorset	2	0	2
Kent & Medway	5	3	8
Sussex	0	1	1
Buckinghamshire, Oxfordshire & Berkshire West & Boundary hospitals	4	6	10

**Table 2:** Characteristics of patients and healthcare professionals interviewed

Patients interviewed (15)	No.
Men Women	5 10
Under 60 60 and over	3 12
Married/living with partner Living alone Carer/dependants	6 7 2
Virtual Clinic Face-to-Face clinic	7 8
TIA TIA mimic Unconfirmed at time of interview	6 8 1
**Healthcare professionals interviewed (12)**	
Consultant grade Specialist nurse Clinic administrator	8 3 1

### Semi-structured qualitative interviews

Two semi-structured interview topic guides – one for patients and one for healthcare professionals – were developed by the research team in collaboration with two Patient and Public Involvement (PPI) project team members with lived experiences of TIA. The topic guides (Appendix 1 and 2) included open-ended questions in the following areas: how care is delivered through virtual/face-to-face/hybrid clinics; the benefits and disadvantages of virtual, face-to-face, and hybrid models of care; resource allocation and environmental sustainability; views and preferences for different models of care; and thoughts on the future of virtual clinics.

Semi-structured qualitative interviews with patients and healthcare professionals were conducted by telephone or Microsoft Teams between February and May 2023. Informed verbal consent was audio recorded at the start of each interview. Participants were sent a copy of the consent form to read before the interview, as well as a copy of the completed consent form afterwards. Interviews lasted for 30–45 min on average and were audio-recorded and transcribed verbatim.

### Data analysis

Interview transcripts were de-identified and entered into NVivo for data management and analysis. Analysis followed a thematic approach, moving from familiarisation and inductive coding to grouping and categorisation [33, 34]. Team ‘data clinics’ explored coding consistency and theme development, using charting, ‘one sheet of paper’ (OSOP) visualisation and constant comparison methods [35] to support the identification and conceptualisation of themes.

## Results

We found that ‘being seen’ by a healthcare professional in a face-to-face consultation strongly mattered to many patients, despite the benefits of virtual consultations that we have discussed in our report [3]. We focused on the benefits of face-to-face consultations in this paper so that we could provide a more in-depth analysis and discussion of why ‘being seen’ in a face-to-face consultation strongly mattered to our patient interviewees, alongside instances where healthcare professionals did or did not agree with the benefits that patients associated with the face-to-face modality. To understand better the components of face-to-face consultations that mattered to patients, we identified and conceptualised two themes:

(1) Greater opportunities for communication (better description of symptoms, being able to ask questions use of non-verbal cues, giving and receiving bad news);

(2) Feeling looked after and being seen by a multidisciplinary team.

### Theme 1: Greater opportunities for communication

#### Better description of symptoms

Face-to-face consultations for TIA were favoured by some patients and clinicians as they enabled better communication between the patient and the healthcare professional. Not all TIA patients felt they could communicate well in a virtual consultation, whether for hearing, language or cognitive difficulties, untimely phone calls or simply a lack of confidence in speaking over the phone. For these patients, face-to-face-clinics enabled (or were perceived to offer) an environment which helped them to describe their symptoms better.

I was out shopping when I got the phone call, so I did feel I was caught off guard a little bit. […] I just think personally a face-to-face consultation would have been better than a phone call. I mean he was asking me lots of questions […] I feel if it was maybe face-to-face, I would have been able to explain it more and they would have listened to, listened to me more. I feel I could’ve explained my symptoms more face-to-face… (TIAP14, diagnosed with TIA mimic, attended virtual clinic)

Clinicians also commented on how a face-to-face clinic benefited them, by enabling access to tacit information about their patients. Some clinicians found taking a history and obtaining an accurate description of symptoms harder over the telephone and considered the face-to-face clinic to be more efficient and effective.

It’s sometimes difficult to make a diagnosis when you are not seeing the patient face-to-face and you cannot examine the patient. You know making a diagnosis is a holistic thing, you need to talk to the patient and get the history, some patients are very poor historians, you know. So they cannot even describe the symptom properly. (TIAS20, Consultant)

Using a virtual model of TIA clinic was seen as especially difficult with patients who have hearing or cognitive impairment.

patients with cognitive impairment and hearing impairment, they will find virtual clinics harder. [… and] it’s harder to use the virtual appointment with them actually, because they always need to have someone [else with them] that you need to go through [in order to speak to the patient], […] and also with those patients actually sometimes you get more information if they are in the clinic, you know, it’s just watching them walking from the door to the chair, how slow they walk, how they stand up, how they move, it can [give you a] little bit of an extra information about what’s happening with them[…] that element of the general examination and clinical examination is not only what they can tell you, but [what] we can see you know so I think it does have an impact actually. (TIAS17, Consultant)

### Being able to ask questions

Patients greatly valued having access to different healthcare professionals, along with multiple opportunities to ask them questions (that might have arisen before or during their appointment) over a longer time period rather than a linear conversation with one healthcare professional.

I did speak to two or three nurses on the team, or doctors, and to actually have a chance to directly speak to the people who are involved, who do work with this all day every day, it’s part of their life, when I come to it completely ignorant, I found [that] a great help. Great help. Very reassuring […] (TIAP25, diagnosed with TIA, attended face-to-face clinic, lives alone)

Patients who were seen in a face-to-face clinic felt that a virtual clinic by telephone would have been less reassuring, limited by a linear conversation with one healthcare professional and lacking in opportunities to reflect or ask questions.

I asked all the questions that I needed to, but I think if I’d have had a virtual appointment I probably wouldn’t have done. […] When you’re actually in an office with them you know that they are there for you. Whereas on the phone they might be having, they might have another patient outside, so you feel a bit more rushed I think. (TIAP23, diagnosed with TIA, attended face-to-face clinic, lives alone)

However, it was possible to create a reassuring virtual clinic experience for patients. Crucially, this required opportunities for patients’ reflections and questions during the consultation but also for healthcare professionals to be responsive to them, creating a reciprocal form of communication. Patients also needed to be prepared for their virtual clinic telephone call, which can be challenging because most virtual TIA outpatient clinic calls are unscheduled due to rapid assessment timelines.

The very next day I had a phone call from the TIA clinic and he was absolutely wonderful, and just talked me through what had happened. I had a phone consultation really of well over half an hour […] he was great. He was just great. It was very clear and I did ask questions. […] and if there was something I didn’t understand I was able to ask and that was good. […] I’d rather just talk on my main phone […] I couldn’t have done this over my mobile if I’d been out and about for example. (TIAP18, diagnosed with TIA mimic, attended virtual clinic, lives alone)

### Use of non-verbal cues

Some patients valued non-verbal aspects of communication to develop rapport, provide reassurance and to impart information.

I personally like the personal touch, so I can understand what the doctor is saying to me and, and he can observe my response. […] I think they could see and feel my response whereas where you’re just on camera, I don’t know that you can pick up those feelings, those personal feelings and observations. (TIAP15, diagnosed with TIA, attended face-to-face clinic, lives alone)

I was happy having face-to-face I have to say, even though it’s a journey because they could see by looking at me that I wasn’t well, you know, when you talk to somebody over the phone it’s a completely different thing. (TIAP23, diagnosed with TIA, attended face-to-face clinic)

This view of the benefits of face-to-face consultations was echoed by some clinicians.

I also, from a, again doctor’s perspective, I find it more satisfying, you have seen your patient face-to-face. You can see their reaction, and I just think there is a better rapport and you can get a better feel for whether they’ve taken whatever you’ve told them on board or not. (TIAS09, Consultant)

On the telephone it’s difficult, isn’t it […] I prefer the face-to-face stuff to be honest because I think you can get a better feel for the patient, they tend to tell you little things that they might have forgotten to mention previously that might, you know, have a bigger impact on what’s happened. (TIAS01, Stroke specialist nurse)

### Giving and receiving bad news

Some clinicians and patients questioned the appropriateness of giving or getting a TIA diagnosis over the phone via a virtual clinic, especially for patients who live alone.

If you’d been told you’d had a TIA, somebody at my age, seventy plus, it’s one of those things you associate with old age, and indeed dying. If I’d have had one I would have liked to have face-to-face a hundred percent. (TIAP04, diagnosed with TIA mimic, attended virtual clinic)

In response to questions on environmental sustainability, some patients who had been seen in a virtual clinic even felt that the environmental benefits of this modality did not outweigh the importance of communicating a TIA diagnosis in a face-to-face consultation.

You can’t always measure it against your carbon footprint. I think if, if it’s something that’s going to affect your whole life, well you know it’s quite important to, to get it done properly. (TIAP08, diagnosed with TIA mimic, attended virtual clinic)

### Theme 2: Feeling looked after and listened to by a multidisciplinary team

For patients that were seen in a face-to-face clinic, the opportunity for interactions with multiple healthcare professionals (rather than just one clinician over the phone) was particularly beneficial. Informal chats while being taken to and from tests, or while waiting in the clinic, reinforced a feeling of reassurance and information-sharing set within the familiarity of a normal routine of going into hospital and ‘being seen’ by multiple people.

I was exposed to the team, who were all very pleasant and very nice people, and, and almost the informal chats that happened as they come and take your temperature or take whatever, […] it felt like a total approach that I think would have been very, very difficult, well impossible on the phone. (TIAP25, diagnosed with TIA, attended face-to-face clinic, lives alone)

Some patients felt that the quality of care during face-to-face clinics was enhanced by multiple healthcare professionals being available to guide them through the process, in addition to continuity of care.

[…] when I received my result it was from the person who initially saw me at the clinic in the morning, who took all the details, as well as, oh I don’t know, probably someone a little bit higher up like a consultant or something, so that felt very reassuring that kind of these two professionals had taken their time to sit down with me and my partner and really covered kind of all bases and I felt like they really had time to listen to me, which is really, like I know how stretched the NHS is at the moment, and I felt quite valued that, they’d given me that amount of time. (TIAP19, diagnosed with TIA mimic, attended face-to-face clinic)

However, whilst this small talk was valuable to the patient experience it was less important for some clinicians, who perceived a decrease in the efficiency of a clinic when using a face-to-face model.

Sometimes you see the patient and within three minutes you’ve got the story, […] you’ve got the diagnosis, but then you feel bad because they’ve already made an effort to come here and the patient might say, ‘Well I came all the way here, and then within five minutes you know everything is over.’ So you might probably extend that conversation a little bit longer to have a little bit of a chinwag with the patient to make them feel that you know their journey to hospital is worth it, […] from my point of view that time is not really adding value. (TIAS17, Consultant)

In contrast, some of the patients who had been seen in a face-to-face ‘one stop shop’ TIA clinic valued its efficiency. Patients could undergo most tests (except for heart monitors) on the same day and be seen by a doctor before they left the clinic. Some test results were also discussed in-person with a healthcare professional, who could formalise a plan of management with the patient. This was a better patient experience that provided greater opportunity to ask questions and gain information, reduce worry, and benefit the emotional wellbeing of patients. There was no need for patients to ‘wait’ and ‘wonder’ when they will be called about their results.

It was nice that […] I didn’t have to then come home and then have a further wait […] I came back and did another couple of hours at work […]. I think if I’d left the clinic without my results I would have felt anxious […] it just made me feel more reassured I guess than a telephone call, and you always worry with a telephone call, is it going to come? What was nice is that I knew when I was walking out that clinic I would know one way or the other the results. (TIAP19, diagnosed with TIA mimic, attended face-to-face clinic)

### Summary

We found that some patients expressed a strong preference for face-to-face clinics for their TIA care, alongside instances where healthcare professionals did or did not express similar preferences. Both face-to-face and virtual clinics for TIA were found to offer some benefits, such as virtual clinics seen as being more time efficient in a few cases. However, both patients and clinicians generally favoured face-to-face consultations for their ability to better support patient wellbeing and care. Some patients felt that the negatives associated with a face-to-face consultation (e.g., the challenges of travel) were outweighed by the benefits, particularly the additional emotional wellbeing and support they experienced. For example, face-to-face clinics provided patients with opportunity to ask more questions and enabled aspects of non-verbal communication that were missing from virtual clinics. Face-to-face clinics also provided patients with a more supportive environment for delivering serious diagnoses, tended to more readily foster continuity of care and allow patients to interact with multiple healthcare professionals which enhanced their sense of feeling cared for.

## Discussion

### Patient and clinician preferences for face-to-face encounters

Much of the current literature highlights convenience for patients as the primary strength of virtual and hybrid models of care. However, for some patients we interviewed the importance of being seen in-person was paramount to any inconvenience which may have arisen from the need to travel to a face-to-face TIA clinic. The perceived seriousness of a suspected TIA, alongside the association of a high risk of stroke following a TIA [32] and the fear of having a potentially life-changing condition, influenced patient preference for face-to-face consultation. Whilst our interviews with healthcare professionals revealed a few cases where virtual TIA clinics were associated with greater efficiency, they have also underscored the difficulties some clinicians experience when conducting virtual assessments for suspected TIA. Face-to-face consultations were preferred by some clinicians in our study, who describe how they could provide more holistic care by being able to better examine particular physical signs and the emotional state of their patients. Therefore, in contrast to existing evidence, our results suggest that when evaluating models of TIA care, the convenience and efficiency of virtual clinics may not always matter most to patients or clinicians.

### Being seen means being heard – the value of presence

Many of our patient interviewees conveyed the importance of ‘being seen’ by a clinician via a face-to-face consultation at some point during their TIA diagnostic pathway. This does not mean that these patients merely wished for a quick and simple consultation or physical examination. Instead, patients want to have opportunities to ask questions and express themselves through non-verbal communication, whilst knowing that they are being listened to and understood by their clinician who responds reassuringly to their queries and concerns. Therefore, when patients emphasised the importance of ‘being seen’ by a clinician, they were implicitly expressing the importance of *being heard* by one, too. In comparison to a virtual consultation, perhaps a face-to-face consultation can therefore be understood to more readily enable a reciprocal process of communication in which patients feel seen and heard. This speaks to the concept of therapeutic presence [36] which suggests that face-to-face interactions enable patients and clinicians to remain more in tune with one another and grounded in the consultation. Given that virtual consultations lack some of the non-verbal aspects of communication that patients and clinicians are accustomed to [37], this is not necessarily surprising. It could however be particularly applicable to patients with a suspected TIA who have hearing, language or cognitive difficulties and find communicating through a virtual clinic to be challenging. 

### TIA clinics and sustainability

Virtual TIA clinics were originally introduced to reduce infection risk during the Covid-19 pandemic. Yet, in the context of a changing climate with evidence of the negative impact of health systems on climate change [38, 39], there is an overarching NHS drive for greater environmental sustainability in healthcare [13, 40]. Within this, virtual care is seen to hold potential for lowering carbon emissions due to reduced patient (and sometimes staff) travel [41]. However, for some patients in our study the potential environmental benefits of virtual care were not outweighed by the importance of being seen in a face-to-face consultation during their TIA care. If environmental sustainability were to become an explicit motive in TIA service planning, the ‘one stop shop’ approach to TIA care that we reported in our results could meet patients’ preferences, whilst also lowering travel related emissions by reducing multiple journeys to and from the TIA clinic. Emissions savings may also be made with a refined referral system that enables clinicians and their teams to better match patient preferences with their suitability to particular TIA clinic modalities.

### Limitations

Our study was part of a rapid insights evaluation and obtaining ethical approval was significantly delayed, shortening our timeframe for recruitment. This meant we were only able to draw our sample from the 6 NHS trusts who agreed to be Patient Identification Centres. We therefore did not have equal spread of patients across the South East Region, but have pockets of experience instead [see Table 1]. Recruitment of healthcare professionals was also compromised by busy clinics, winter pressures and strike action. As a result, our participant sample was much smaller and less diverse (e.g., double the number of women than men in the patient participant sample) than our recruitment target of 55 participants, with approx. 30 patients/carers and 24 healthcare professionals (10 patients/carers and 8 healthcare professionals per TIA care modality). Further research is therefore needed to understand whether the experiences and views expressed by our interviewees are representative of wider and more diverse populations. Future research could use mixed methods study designs to understand meanings behind patterns in experiences of/views on different TIA clinic modalities from larger and more diverse patient and healthcare professional samples. For even richer qualitative insight, observational research of patient and healthcare professional interactions across the TIA diagnostic pathway and different clinic modalities could generate data that can be triangulated with interview data.

## Conclusions

This small-scale rapid evaluation highlights that face-to-face consultations are significant for some patients with a suspected TIA, who expressed that ‘being seen’ in a face-to-face consultation by a clinician greatly mattered to them. Whilst virtual clinics offer certain advantages, such as time-efficiency and potentially reduced carbon emissions, our findings suggest that some patients and healthcare professionals may place greater value on the benefits of face-to-face consultations instead. Our study sample size is small but our results offer rich insights. Therefore, whilst recognising further research is needed, we propose that our study insights offer some direction for thought about how TIA services are planned and delivered. This could include investigating whether a hybrid model of TIA care may be able to offer the greatest potential benefits to patients and healthcare professionals. We suggest patient choice of TIA care modality would be key to support this, as well as a refined referral system that enables healthcare professionals to better appreciate which type of patient would be best for virtual and face-to-face care. Better communications skills training for healthcare professionals delivering virtual care could also help. Future TIA service planning and delivery efforts could focus on refining care models that prioritise patient-clinician relationships, whilst also considering the broader implications for sustainability and accessibility in healthcare.

## Supplementary Information

Below is the link to the electronic supplementary material.

Supplementary Material 1

Supplementary Material 2

## Data Availability

Data is securely stored in University of Oxford data storage and not available with permissions or for sharing due to information governance and ethics restrictions.

## Abbreviations

A&E: Accident and emergency department
NHS: National health service
TIA: Transient ischaemic attack
MRI: MRI imaging magnetic resonance imaging
MRA: MRA magnetic resonance angiography
ISDNs: ISDNs integrated stroke delivery networks
GRIFT: Getting it right first time
PERCS: Planning and evaluating remote consultation services framework
NIPP: NHS England’s insights prioritisation programme
AHSN: Academic health science network
ARC: Applied research collaboration
PPI: Patient and public involvement
OSOP: One sheet of paper
