# Supplementary material for: The importance of “being seen”: a qualitative interview study of patient and clinician experiences of virtual and face-to-face TIA clinics in one region of England
Source: BMC Health Serv Res. 2026 Sep 14;26:1314. doi: 10.1186/s12913-026-15470-6 (PMC13615589; doi:10.1186/s12913-026-15470-6)
Supplement: Supplementary file 1 [file 12913_2026_15470_MOESM1_ESM.pdf]

HEALTH CARE PROFESSIONALS INTERVIEW TOPIC GUIDE

Introduction

- Check participant information leaflet has been received and read. Including explanation on the limitations on the confidentiality with regards to safeguarding issues.
- Explain purpose of the interview – to find out healthcare professional's views and experiences of virtual and hybrid TIA clinics
- Any questions?
- Take consent and start recording

Interview

1. To begin with please can you tell me what your current job title is and your role in the TIA/stroke service?
2. Can you explain how TIA care is currently provided in your local area?

Probe:

- Face to face/virtual/hybrid
- Pre-pandemic/ changes in and since the pandemic
- What is the role of the TIA service?

3. Why were virtual TIA clinics introduced in your local area?

4. What were the difficulties (if any) in setting up virtual TIA clinics?

5. Have any changes been made to the way TIA clinics are delivered since they were set up?

6. How far are virtual clinics now embedded as 'usual care'?

Now thinking about how TIA care is delivered through virtual clinics.

7. What does 'virtual' mean/encompass?

Probe:

- A phone call, or Zoom/Teams/Facetime? ○
- How does 'virtual' work at triage, consultation and follow-up?
- How are TIA patients identified and referred, and by whom?
- How is virtual assessment conducted?

8. How are resources used - e.g., workforce configuration and time utilization for virtual clinics?

Probe:

- Is this better or worse using virtual delivery? Why?
- Has environmental sustainability/carbon footprint featured in discussions about the shift to virtual? How has this shaped service design/delivery?
- What journey would you usually make for face to face clinic/appointments, and how has this changed with the shift to virtual? (N.B. looking here for distance and mode of travel – car, bus, train etc.,)

9. **If stopped:** Why were virtual clinics stopped in your local area?

10. In your opinion, which aspects of TIA care provision work well virtually? Why?

    - Efficiencies/ better resource use

    - Impact on DNA rates

11. Which aspects of TIA care provision do not work well virtually? Why?

    - What (if any) are the risks?
    - What (if anything) gets missed/neglected?

12. Are there any patients for whom delivering TIA care virtually is less appropriate? Probe:

    - Access to technology/broadband reliability/social inequalities
    - Age of patient
    - Where communicating with healthcare professionals may be difficult/interpreter needed/cognitive constraints/confidence
    - Clinical reasons

13. How do you assess how well the virtual clinics are working?

14. How do virtual clinics support/prevent holistic care? Probe:
    - Aligned with what the patient wants and addresses all concerns – both physical and emotional
    - See the bigger picture not just a diagnosis
    - Addresses practical concerns, such as location of care (nearer home?), transport issues
    - Timing of diagnostic tests supports patients physical and emotional needs

    - Enables/prevents support from wider support/services (emotional and physical)

- Offers secondary prevention and behavior change ○ Enables periodic reviews'- e.g., after 3 months initially, 6 months, one year.

15. Have you received any feedback from patients or their family/carers about the virtual clinics? Probe:
    - Appropriate/better/worse/like/dislike? Why?

16. Have you received any feedback from other healthcare professionals about the virtual clinics?

    Probe:
    - List other staff roles and ask if appropriate/better/worse/like/dislike – Why?

17. Now thinking about the future development of your TIA services, what plans do you have? Probe:
    - How do you see them progressing?
    - Where does environmental sustainability and issues of decarbonisation fit in? (i.e. environmental sustainability and potential for decarbonisation through use of virtual clinics (due to changes in travel, use of fossil fuel, reduced emissions etc.).
    - What are the potential other benefits and disbenefits of virtual services going forward? (facilities/resources needed, travel and time trade-offs, fit with other work practices

18. What might help delivery of virtual clinics in the future?

19. Finally, do you have any advice/comments you can give us about change/improvements to virtual clinics?

<u>Close</u>

○ Check for any final questions, points, reflections

○ Thank for participation

○ Explain the next steps in this study
