# Supplementary material for: The importance of “being seen”: a qualitative interview study of patient and clinician experiences of virtual and face-to-face TIA clinics in one region of England
Source: BMC Health Serv Res. 2026 Sep 14;26:1314. doi: 10.1186/s12913-026-15470-6 (PMC13615589; doi:10.1186/s12913-026-15470-6)
Supplement: Supplementary file 2 [file 12913_2026_15470_MOESM2_ESM.pdf]

PATIENT/CARER INTERVIEW TOPIC GUIDE

Introduction

- Check participant information leaflet has been received and read. Including explanation on the limitations on the confidentiality with regards to safeguarding issues.
- Describe what a transient ischemic attack (TIA) is.
- Explain what virtual TIA clinics are including the context in which they started and how they differ from face to face.
    - Explain what a hybrid approach means and what practical arrangements are involved.
    - Explain purpose of the interview – to find out patient's views and experiences of face to face, virtual and hybrid TIA clinics.
    - Any questions?
    - Obtain consent and start recording.

Interview

We are interested in TIA (s) that you have experienced since March 2020.

1. Can you start by telling me about the TIA (s) you have experienced since March 2020.

   Probe:
    - What were your symptoms?
    - How did you recognise that you had experienced a TIA?
    - What did you think might be the cause of your symptoms (if anything)?
    - Establish any previous experiences of face-to-face TIA clinic care.

2. Which healthcare provider did you contact first?

   Probe:
    - GP, A&E, Ambulance service, NHS 111?
    - Who made the contact? (patient, patient's family member, carer)

3. How has the care you received for your TIA been provided - virtually or face to face or a mix of both?

   Probe:
    - Experiences of referral
        - Initial triage with doctor/nurse/paramedic
        - Doctor consultation
        - Tests/investigations
        - Follow-up?

4. Can you tell me about the diagnostic tests you had.

   Probe:

- How long did you wait to have diagnostic tests?
- Which ones and where?
- Did you understand what they were for, and what the concerns were?
- How did you feel about having the tests in a one stop visit/spread over several visits? Why?

5. Thinking now about the timescales of receiving healthcare for your TIA, how did you feel about timescales from first seeking help to returning home?

Probe: first contact, transfers, waits to be seen, discharge -TIA to returning home?

Now I'd like to find out more about your views on receiving care through a virtual/face to face/hybrid model of care (particularly what you thought about the virtual aspect of your care).

Virtual experiences:

6. What did you like about being seen virtually?

    Probe:

    - Location – travel time, transport issues – what distance would you have travelled for a face-to-face appointment, and by what form of transport? (N.B. looking here for distance and mode of travel – car, bus, train etc.,
    - Speed of receiving care
    - Convenience- saves time/money through needing to take time off work or arrange cover for caring commitments
    - Felt confident in communicating with healthcare professionals through technology
    - Comfortable being seen in home environment
    - Any other benefits, e.g., reducing carbon emissions through reduced travel / supporting a greener NHS

7. What did you dislike about being seen virtually?

    Probe:

    - Prefers in person consultations (worry that something might be missed)
    - Technology – access or phone internet connection problems
    - Communicating with healthcare professionals/interpreter needed/cognitive constraints/confidence
    - Timing – appointments not available when quiet space at home
    - Other limiting conditions
    - Any other concerns

Face to face experiences:

8. What did you like about being seen face to face?
   Probe:
   - Prefers communicating by in person consultations
   - Reassurance of seeing someone
   - Speed of receiving care
   - Any other benefits

9. What did you dislike about being seen face to face?
   Probe:
   - Location – travel time, transport issues, parking costs and accessibility
   - Speed of receiving care
   - Time off work, cover for caring commitments
   - Any other concerns

10. Were there any parts of your TIA healthcare experience which you felt worked particularly well through a virtual or face to face consultation? Why?
    Probe:
    - Triage
    - Consultation
    - Follow up appointments

11. How were your concerns or worries about your TIA addressed at the virtual/face to face appointments?
    Probe:
    - How reassured did you feel? Why?

12. Thinking now about your knowledge of TIA and stroke, how much did you know about stroke and stroke symptoms <u>before</u> having a TIA?

13. After your TIA, how did healthcare professionals give you information about the symptoms of stroke and what to look out for?
    Probe:
    - Written/online versus verbal information
    - When was the information provided – after receiving care/medication or very soon after the initial TIA referral?
    - Was this an appropriate way of receiving information/enough information?
    - Were you given any information about prevention/behaviour change?

14. Healthcare professionals want to make sure that they are meeting patients' needs which is sometimes called 'holistic care'. What does holistic care mean to you, what would need to be provided?

    Probe:

    o Aligned with what the patient wants and address all concerns – both physical and emotional

    - Health professionals see the bigger picture not just a diagnosis
    - Addresses practical concerns, such as location of care (nearer home?), transport issues
    - The timing of diagnostic tests support patients physical and emotional needs
    - Enables support from wider support/services (emotional and physical)
    - Offers secondary prevention and behavior change
    - Periodic reviews- e.g., after 3 months initially, 6 months, 1 year
    - Periodic check-ins initiated by the GP practice (Blood tests)

15. Finally, if TIA clinic appointments were to be run virtually in the future how would feel about this?

    Probe:

    o Better/worse/no difference than face to face

    - Any advice/comments you can give us about change/improvements to virtual clinics
    - Future use of virtual clinics as a way of helping to develop a greener NHS?

<u>Close</u>

o Check for any final questions, points, reflections

o Thank for participation

o Explain the next steps in this study
